# Is it possible to reduce the rate of vertical transmission and improve perinatal outcomes by inclusion of remdesivir in treatment regimen of pregnant women with COVID–19?

**DOI:** 10.1186/s12884-023-05405-y

**Published:** 2023-02-13

**Authors:** Nader Tavakoli, Shahla Chaichian, Jamileh Sadat Sadraei, Saeedeh Sarhadi, Sepideh Arbabi Bidgoli, Elnaz Rokhsat, Katayoon Anoushirvani, Banafsheh Nikfar, Abolfazl Mehdizadehkashi

**Affiliations:** 1 Trauma and Injury Research Center, Iran University of Medical Sciences, Tehran, Iran; 2 Endometriosis Research Center, Iran University of Medical Sciences, Tehran, Iran; 3 Iranian Society of Minimally Invasive Gynecology, Tehran, Iran; 4 Health Promotion Research Center, Zahedan University of Medical Sciences, Zahedan, Iran; 5 Department of Community Medicine, School of Medicine, Zahedan, Iran; 6 Department of Toxicology and Pharmacology, Faculty of Pharmacy and Pharmaceutical Sciences, Islamic Azad University, Tehran Medical Sciences University, Tehran, Iran; 7 Pars Advanced and Minimally Invasive Medical Manners Research Center, Pars Hospital, Iran University of Medical Sciences, Tehran, Iran

**Keywords:** COVID–19, Pregnant women, Remdesivir, Neonatal, Vertical transmission, Pregnancy outcome, Perinatal

## Abstract

**Background:**

Coronavirus disease 2019 (COVID–19) is currently one of the world's most critical health issues so far. Given the importance of appropriate treatment in pregnancy and the controversies about Remdesivir effectiveness and complications, the present study aimed to evaluate the impact of Remdesivir on maternal, fetal, and perinatal outcomes in pregnant women with COVID–19 diseases.

**Methods:**

A total of 189 pregnant women with positive polymerase chain reaction (PCR) results for SARS–COV–2, and oxygen saturation [SpO2] of < 95%) were admitted to 12 hospitals affiliated with the Iran University of Medical Sciences from March 1^st^, 2020 to June 7^th^, 2021, namely the first four COVID-19 Picks in Iran. They were enrolled in this retrospective cohort study by census method and categorized into case and control groups, based on the inclusion of Remdesivir in their treatment protocol. Demographics, clinical outcomes, and pregnancy-related complications of the mothers and the neonates were compared between the two study groups.

**Results:**

A comparison of 54 mothers in the case and 135 in the control group showed no demographic and clinical characteristics difference. Neonates whose mothers did not receive Remdesivir had a higher rate of positive PCR (10.2%), compared to the Remdesivir group (1.9%) with a relative risk of 0.91 reported for Remdesivir (95% CI: 0.85–0.98, *P* = 0.04); besides, Remdesivir resulted in fewer neonatal intensive care unit admission rates in mild/moderate COVID–19 group (RR = 0.32, 95% CI: 0.105–1.02, *P* = 0.03). Although neonatal death between the two groups was not statistically significant, from the clinical point seems important; 1(1.9%) in the case vs. 9(7.2%) in the control group. Interestingly LOS (Length of Stay) in the hospital was longer in the case group (median of 7 vs. 3 days; *P* < 0.0001).

**Conclusion:**

The inclusion of Remdesivir in the treatment protocol of pregnant women with COVID–19 may reduce vertical transmission and improve perinatal outcomes, thus being suggested to be considered.

**Supplementary Information:**

The online version contains supplementary material available at 10.1186/s12884-023-05405-y.

## Introduction

Coronavirus disease 2019 (COVID–19) is one the most critical health issue of the twenty-first century, resulting in nearly 6.4 million death so far. Despite acceptable adherence to health protocols, social distancing regulations, the use of various treatment modalities, and the innovation of a new generation of vaccines, the pandemic is still ongoing.

Although it seemed that the complications and morbidity and mortality were not significantly higher than the normal populations in the first few months of the pandemic [1, 2], as time passed maternal morbidity and mortality, adverse pregnancy outcomes and vertical transmission attracted more attention. Even it seems that with new mutants of the virus, these adverse effects are now more prevalent [3]. Takemoto et al. have demonstrated that the mortality rate in pregnant women with Covid-19 disease was about two times higher in 2021 (15.6%), in comparison with 2020 (7.4%) [4, 5].

Considering pregnant women in research projects for finding better therapeutic modalities and creating balanced and informed evidence-based data on this very insidious disease is critical.

Remdesivir is a nucleotide analog with broad-spectrum antiviral activity, that is thought to inhibit the action of RNA polymerase.

In vitro studies have shown that this adenosine analog incorporates nascent viral RNA chains, resulting in premature termination and inhibition of viral replication with a high selectivity index [6–8]. Animal studies have also confirmed its efficacy in reducing the viral load in bronchoalveolar lavage fluid and attenuated pulmonary infiltrates [9, 10]. The few clinical studies about the effectiveness of Remdesivir have suggested clinical improvement, shorter recovery time, lower oxygen requirement, and mortality rate [11, 12]; however, some trials have rejected significant expediency of Remdesivir on clinical outcomes [13, 14]. Therefore, ongoing clinical trials are being conducted to determine the Remdesivir efficiency in the different subgroups of patients [15].

Despite conditional recommendation against its prescription, Remdesivir could still be effective in early mortality risk reduction and clinical improvement, as well as decreased high-flow supplemental oxygen and invasive mechanical ventilation necessities among hospitalized COVID-19 patients [16]. However, recent reports on Remdesivir-related acute kidney injury as well as its cardiotoxicity concerns by a significant decrease in oxygen consumption, a collapse of mitochondrial membrane potential, and an increase in lactate secretion according to an in vitro study [17] is a call for a better characterization of Remdesivir safety, understanding of the underlying mechanisms and suggesting a careful evaluation of therapeutic use in patients at risk for cardiovascular or kidney disease.

It is not overemphasized if somebody believes that pregnant women are among the most important groups. Besides, pregnancy accounts for a modified immune and inflammatory estate that may contribute to the activation of inflammatory pathways at the time of the Covid-19 trigger [18].

COVID–19 diseases in this group can endanger the mother’s life, threaten fetal well-being, transmit to the newborn, and result in complications and mortality in the mother and the neonate [19–22]. Summarizing evidence regarding the use of Remdesivir as an investigational pharmacologic intervention for pregnant and lactating patients with coronavirus disease 2019 (COVID-19) is controversial, but it has been used during pregnancy and lactation within the context of clinical trials, data from non-pregnant populations have not shown benefit [23].

Given the importance of appropriate treatment in patient recovery and the controversies about Remdesivir, the present study aimed to evaluate the efficacy of Remdesivir in the treatment regimen of pregnant women with COVID–19 on their recovery and perinatal outcomes including PCR test results (vertical transmission), NICU admission and neonatal mortality.

## Methods

The present retrospective cohort study considered pregnant women with COVID–19, admitted to 12 hospitals affiliated with the Iran University of Medical Sciences, from March 1^st^, 2020 to June 7^th^, 2021 for inclusion. Of 39,746 deliveries in the time and setting, 189 mothers had the inclusion criteria (4.7 per 1,000 pregnant women). The clinical guideline for diagnosing and treating COVID–19 diseases issued by the Iran Ministry of Health and Medical Education were used in the present study. Diagnosis of COVID–19 was suspected by clinical symptoms and confirmed by polymerase chain reaction (PCR) results, taken by nasopharyngeal swab, and in suspicious cases by chest CT taken with a protective shield. An infectious specialist and a gynecologist admitted the patients based on SpO2 of 93–95%. The ethics committee approved the protocol of the study at the Iran University of Medical Sciences (code: IR.IUMS.REC.1400.514).

**Fig. 1:**
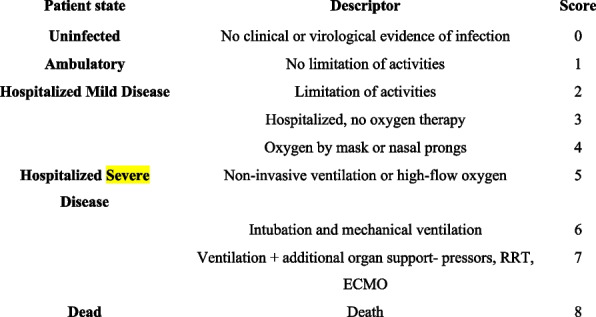
Ordinal Scale for Clinical Improvement (WHO R&D Blueprint) [1]

A total of 189 women with the above criteria were enrolled in the study by census method and categorized into case and control groups, based on the inclusion of There is no unique treatment protocol that can be used for all patients with COVID–19 and the therapeutic options depend on the patient’s conditions. Patients who have a low oxygen saturation (SpO_2_) are managed in the hospital with a combination of anti-inflammatory agents, such as corticosteroids and monoclonal antibodies, i.e. Tocilizumab, which counts as the standard of care in Covid-19 patients [24], antiviral agents, and interferons in some cases [25]. Remdesivir (GS–5734) is a broad-spectrum antiviral drug, approved by the Food and Drug Administration (FDA) as the emergency treatment of COVID–19 [26, 27]. Remdesivir was administered 100–200 mg/day for 5–10 days based on the patient’s general conditions and response to treatment. The patients remained in the hospital until the completion of the Remdesivir course. All patients received the standard of care of COVID–19 protocol according to WHO living guidelines [24] for pregnant women. According to the ordinal scale for clinical improvement by WHO (Fig. 1). We recruited the patients by scoring three severity at the time of admission.

A midwife and one general physician evaluated the medical records independently and recorded their information in the study’s checklist; then, another physician compared the recorded data with the medical records to ensure the accuracy of the collected data. The recorded information (in addition to the type of treatment) includes baseline characteristics (maternal age, weight, and height at admission), body mass index (BMI), the clinical outcomes, including LOS and ICU, readmission, intubation, cardiopulmonary resuscitation (CPR), death, and its cause, and finally pregnancy-related complications, such as pre-eclampsia, preterm labor, etc.

The LOS in the hospital and ICU were recorded separately and LOS in the hospital was calculated as the entire period of the patient’s hospital stay, including the ward and the ICU. Patients were discharged at the end of the Remdesivir course. Any patient readmitted after discharge was considered as readmission. Patients who did not recover from COVID–19 and died as a result of the disease within one month after diagnosis were considered COVID–19–related death. Cases that died of other causes, such as accidents and underlying conditions, were not considered COVID–19–related death and were excluded from the analysis. The causes of death were categorized into acute respiratory distress syndrome (ARDS) and non–ARDS. The criteria of ARDS were confirmed based on the Berlin 2012 ARDS diagnostic criteria [28].

We evaluated the pregnancy outcomes, including abortion, preterm or post-term pregnancy, neonatal PCR result, admission to NICU, and neonatal mortality. The test was considered positive if PCR results of 24 h after birth or at the second test 48 h after delivery were positive (in the case of a negative first test result), deaths were considered neonatal death during the first 28 days of life, excluding other pathologies. For the follow–up information, the mothers or their relatives were contacted by phone call and related information was recorded. Some who did not remember the details or did not replied the phone or had missing data in their medical records that could not be completed by the following–up phone call, were considered missing data.

### Statistical analysis

The qualitative data were presented as a percentage (frequency of observation) and compared using Chi-square or Fisher’s exact test. For numeric variables, the Kolmogorov–Smirnov test was performed and if the data were normally distributed, mean and standard deviation (SD) were reported, and if skewed, median and interquartile range (IQR) were reported. The student *t-test* or Mann–Whitney U test was used for comparison between the groups, based on the normality of data distribution. The estimated effects were expressed as a relative risk (RR) with a 95% confidence range (CI). *P*–values < 0.05 were considered significant. The analysis was conducted using the statistical software IBM SPSS Statistics for Windows version 22.0 (IBM Corp. 2013. Armonk, NY: IBM Corp.)

## Results

**Fig. 2:**
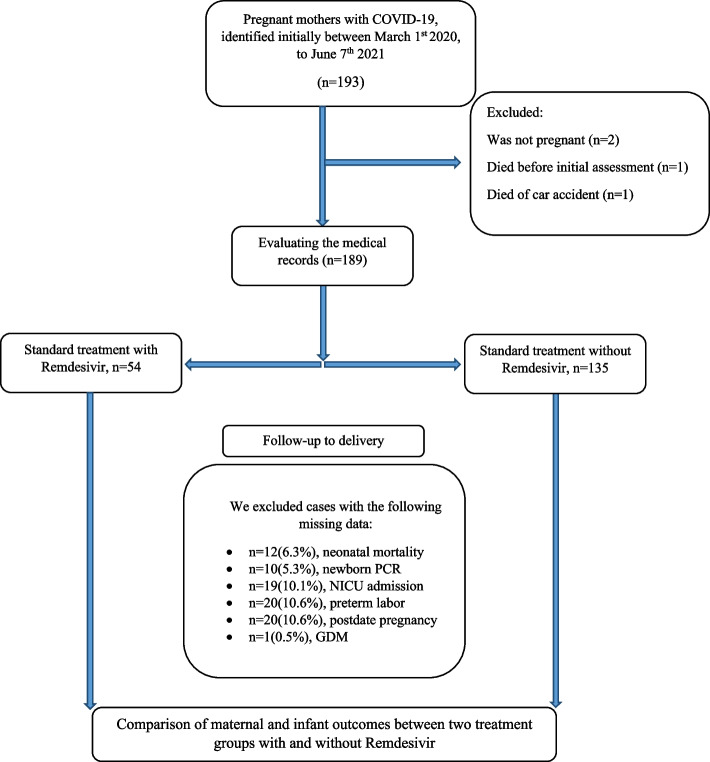
Flow chart of pregnant women with COVID-19 in cohort study

Among 189 mothers (with a median age of 32 ± 7 years), 54 were in the case and 135 were in the control group (Fig. 2). There was no significant difference in age, BMI, and other baseline or pregnancy-related characteristics between the groups (Table 1). A comparison of the complete demographics of the groups is provided in Supplementary Table 1. COVID–19 symptoms mainly started at a mean ± SD of 32.7 ± 7.1 weeks gestational age; namely in the third pregnancy trimester (*N* = 162; 85.5%); there was no difference in the frequency of the trimester that the symptoms started between the two study groups (*N* = 0.532, Table 1). A comparison of the maternal outcomes between the two groups with and without Remdesivir in the treatment protocol (Table 1) showed longer LOS in the hospital in the Remdesivir group (*P* < 0.0001). Other maternal outcomes, like the need for ICU admission, intubation, CPR, and COVID–19–related death, were not different between the groups (*P* > 0.05).

**Table 1:** Comparison of mother’s demographics, pregnancy-related, and clinical characteristics based on inclusion of Remdesivir in the treatment regimen

Variable	Categories	Total N	Remdesivir *N* = 54	Non–Remdesivir *N* = 135	*p*–value
**Age (years), median(IQR)**		189	32(6)	32(7)	0.924^*^
**BMI (kg/m2), median (IQR)**		189	28(5.5)	28(6)	0.210^*^
**BMI, Categories, N(%)**	**Normal**	189	9(16.7%)	30(22.2%)	0.695^†^
	**Overweight**		24(44.4%)	56(41.5%)	
	**Obese**		21(389%)	49(36.5%)	
**Gravid, median (IQR)**		189	2(1.25)	2(2)	0.07^*^
**Abortion, median (IQR)**		189	0(1)	0(1)	0.78^*^
**Preeclampsia, N(%)**		189	2(3.7%)	15(11.1%)	0.08^a^
**Gestational diabetes, N(%)**		188	8(14.8%)	19(14.25%)	0.91^b^
**Abortion, N(%)**		179	3(5.6%)	7(5.2%)	0.58^a^
**Gestational week at the onset of COVID–19 symptoms (weeks), N.(%)**	First Trimester	189	3(5.6%)	4(3%)	0.532^†^
	Second Trimester		7(13%)	13(9.6%)	
	Third trimester		44(81.5%)	118(87.4%)	
**First SPO**_**2**_** without a mask, N.(%)**	< 94	43	15(27.8%)	28(20.7%)	0.29^†^
	≥ 94	146	39(72.2%)	107(79.3%)	
**Severe COVID–19, N(%)**		189	15(27.8%)	28(20.7%)	0.33^†^
**Admission to ICU, N(%)**		189	5(9.3%)	20(14.8%)	0.308^†^
**Intubation, N(%)**		189	2(3.7%)	11(8.1%)	0.27^†^
**Cardiopulmonary resuscitation, N(%)**		189	2(3.7%)	7(5.2%)	0.49^‡^
**COVID–19–related death, N(%)**		189	3(5.6%)	10(7.4%)	0.46^‡^
**ARDS as the cause of death**		189	0(0%)	5(3.7%)	0.18^‡^
**Hospital LOS (days), median (IQR)**		189	7(4)	3(4)	< 0.0001^*^
**ICU LOS (days), median (IQR)**		25	4(5.5)	3(6.75)	0.65^*^

*Abbreviations*: *IQR* Interquartile range, *BMI* Body mass index, *COVID–19* Coronavirus disease 2019, *SPO*_*2*_ Saturation of peripheral oxygen, *ICU* Intensive care unit, *ARDS* Acute respiratory distress syndrome

^*^The results of Mann Whitney U test, ^†^The result of chi-square test, ^‡^The result of Fisher’s exact test

Among all patients, 146 (77.2%) had mild or moderate and 43 (22.8%) had severe COVID–19. Studying the difference between the two groups, considering the disease severity, showed no difference in most variables (*P* > 0.05), the case group had a longer LOS (median of 7 days) than the control group (*p* < 0.001)*.* However, this discrepancy may be due to the mandatory hospital stay for pregnant women under antiviral agents, even after symptom relief.

A Comparison of neonatal outcomes between the two study groups (Table 2) showed no difference in most of the studied variables, except in the positive PCR results. Neonates whose mothers did not receive Remdesivir had a higher rate of positive PCR (10.2%), compared to the Remdesivir group (1.9%) with a relative risk of 0.91 reported for the case group (95% CI: 0.85–0.98, *P* = 0.04).

**Table 2:** Comparison of neonatal outcomes between the two study groups, in total and based on oxygen saturation

Variables	Categories	Total N	Remdesivir *N* = 54	Non–Remdesivir *N* = 135	Relative risk (95% confidence interval)	*p*–value
**Preterm labor, N(%)**	**Total**	169	18(36.7%)	45(37.5%)	0.98(0.63–1.51)	0.92^*^
	**SPO**_**2**_** ≥ 94%**	130	13(36.1%)	33(35.1%)	1.02(0.61–1.72)	0.91^*^
	**SPO**_**2**_** < 94%**	39	5(38.5%)	12(46.2%)	0.83(0.37–1.86)	0.64^*^
**Postdate pregnancy, N(%)**	**Total**	169	1(2%)	7(5.8%)	0.35(0.44–2.76)	0.26^†^
	**SPO**_**2**_** ≥ 94%**	130	0(0%)	6(5.6%)	–^‡^	0.13^†^
	**SPO**_**2**_** < 94%**	39	1(7.7%)	1(3.8%)	2(0.13–29.4)	0.56^†^
**NICU admission, N(%)**	**Total**	170	7((13.7%)	30(25.2%)	0.54(0.2–1.15)	0.09^*^
	**SPO**_**2**_** ≥ 94%**	134	3(8.1%)	24(24.7%)	0.32(0.105–1.02)	0.03^*^
	**SPO**_**2**_** < 94%**	36	4(28.6%)	6(27.3%)	1.04(0.35–3.06)	0.61^†^
**Neonatal death, N(%)**	**Total**	177	1(1.9%)	9(7.2%)	0.26(0.35–2.05)	0.15^†^

*Abbreviations*: *SPO*_*2*_ Saturation of peripheral oxygen, *NICU* Newborn intensive care unit, *PCR* Polymerase chain reaction test

^*^The results of Chi square test, ^†^The result of Fisher’s exact test, ^‡^One cell of 2 × 2 table has observed count 0; therefore, RR is not computable

Also, Remdesivir resulted in fewer NICU admission in neonates of mothers with mild/moderate COVID–19 (RR = 0.32, 95% CI: 0.105–1.02, *P* = 0.03).

## Discussion

The present study is the first to evaluate the effect of the inclusion of Remdesivir on maternal and perinatal outcomes of pregnant women compared to the control group. The comparison between the two (case and control) groups with similar demographic and clinical characteristics showed no difference in most of the maternal outcomes, except LOS; however, the most important finding of the present study was the lower risk of vertical transmission of COVID–19 to the infant, and fewer neonatal intensive care unit admission rates in mild/moderate COVID–19 group and clinically important less neonatal death which is of great significance.

Evidence has suggested that pregnant women are not more susceptible to COVID–19; however, they are exposed to a higher risk of adverse outcomes, which indicates the importance of studying COVID–19 outcomes in pregnant women [29]. Neonatal health is one of the aspects that has been rarely discussed in this regard. As the present study results showed, 7.8% of the infants (14/179), born to mothers admitted with COVID–19, had positive PCR results. This rate is similar to that reported by the meta-analysis of 33 studies, reporting this rate at 6.3% (95% CI of 3.0 to 9.7%) [30]. In another (cohort) study on 706 pregnant women with COVID–19, compared with 1,424 controls, 13% of the neonates tested positive for COVID–19 [31], which is higher than the rate in our study. This difference could be related to neonatal PCR testing protocols. However, it is not yet clearly defined how it can affect neonatal health, although a higher rate of neonatal death and stillbirth has been observed during the pandemic than the pre-pandemic rates [32, 33]. However, this cannot be directly related to vertical transmission, as COVID–19 on increasing the pregnancy-related adverse effects, and the reduced referral of pregnant women to the care centers could also be contributing factor [34]. Therefore, further studies are required to confirm the effect of vertical transmission of COVID–19 on neonatal health. In the present study, all neonates who tested positive in the control group had SpO_2_ ≥ 94% with mild to moderate COVID–19 observed in their mothers, but the one neonate in the case group had SpO_2_ < 94%. The present study results determined (for the first time) that the inclusion of Remdesivir in the treatment protocol of mothers with COVID–19 reduces its vertical transmission (*P* = 0.03). Also, Remdesivir resulted in fewer NICU admission when mothers were involved with mild/moderate COVID–19 (RR = 0.32).

Most studies evaluating the effect of Remdesivir on COVID–19 have excluded pregnant and lactating women and there are few studies on the effect of Remdesivir on the outcomes of pregnant women with COVID–19 [35]. In one study on 86 pregnant and postpartum women with severe COVID–19, compassionate–use of Remdesivir resulted in a high recovery rate and low rate of serious adverse events [36]; however, this study had no control group for comparison. Although we could not find a cohort study with a control group for comparing the results with our research, it should be acknowledged that the different rates of efficacy and adverse outcomes, reported in the studies may be related to the different patients’ conditions. Disease severity is one of the factors that have a significant influence on the outcome of COVID–19; in the present study, less than one–third of each group had severe disease, while the cohort by Burwick et al. included pregnant women with severe COVID–19, which justifies the differences in the rates of adverse outcomes. Other case reports/series have also reported the efficacy of Remdesivir in pregnant women with COVID–19 [37–41], a review of which suggests that Remdesivir is well-tolerated in the latter stages of pregnancy (2^nd^ and 3^rd^ trimesters) with a low risk of serious adverse events; however, further safety and efficacy data are required to support its use in the first trimester [42].

A Comparison of the adverse pregnancy-related and COVID–19 outcomes did not show any significant difference between the groups, suggesting that Remdesivir cannot reduce the risk of maternal effects. However, it has to be mentioned that the rate of adverse pregnancy-related and COVID–19 outcomes was generally low in the present study, compared to the previous studies, which could be related to the effect of disease severity on the adverse pregnancy-related and COVID–19 outcomes in pregnant women, as suggested by the meta-analysis of 44 studies (438,548 pregnant women with COVID–19) [21]. The only maternal outcome with a statistically significant difference between the study groups was the median LOS, longer in the case group. The primary explanation for this difference could be the mandatory hospital stay of pregnant women during the antiviral treatment course. However, this finding could be related to the confounding effect of different variables on the study outcomes. We acknowledge that the difference in the treatment protocol of the patients (selected based on the clinical conditions of the patients and the national protocols) and the different disease severities among patients may confound the study results and serve as a limitation. Also, the study's limited sample size and non–randomized inclusion of patients into the research and subgroups was another limitation of the present study.

## Conclusion

The results of our research clearly defined that the inclusion of Remdesivir in the treatment protocol of pregnant women with COVID–19 can reduce the vertical transmission rate, NICU admission, and probably neonatal death. Considering the limitations of the present study, if future studies confirm these results, Remdesivir can be suggested as a safe and efficient medication for this group of patients. However, the results of our study failed to show any effect of Remdesivir on the adverse pregnancy-related and COVID–19 outcomes of pregnant women and even prolonged the LOS in the hospital. As there are very few studies addressing the maternal outcomes of COVID–19, none comparing the results with a control group, a future study with a focus on the more detailed pregnancy outcomes is required for a definite conclusion in this regard.

## Supplementary Information

**Additional file 1:** **Supplementary Table 1. **Comparisonof demographic characteristics of pregnant women with COVID–19 between Remdesivirand non–Remdesivir groups.

## Data Availability

The datasets generated and/or analyzed during the current study are not publicly available due to the author's sensitivity but are available from the corresponding author upon reasonable request.
