# Supplementary material for: Is it possible to reduce the rate of vertical transmission and improve perinatal outcomes by inclusion of remdesivir in treatment regimen of pregnant women with COVID–19?
Source: BMC Pregnancy Childbirth. 2023 Feb 13;23:110. doi: 10.1186/s12884-023-05405-y (PMC9923661; doi:10.1186/s12884-023-05405-y)
Supplement: Supplementary file 1 — Additional file 1: Supplementary Table 1. Comparisonof demographic characteristics of pregnant women with COVID–19 between Remdesivirand non–Remdesivir groups. [file 12884_2023_5405_MOESM1_ESM.docx]

**Supplementary Table 1. Comparison of demographic characteristics of pregnant women with COVID–19 between Remdesivir and non–Remdesivir groups**

| **Variable** | **Categories** | **Total N** | **Remdesivir group (N=54)** | **Non–Remdesivir group (N=135)** | **P-value** |
| --- | --- | --- | --- | --- | --- |
| **Weight (kg), median(IQR)** | | 189 | 76.5(18) | 75(16) | 0.283^*^ |
| **Height (cm), mean±SD** | | 189 | 163.3±5.4 | 163.2±5.5 | 0.864^†^ |
| **Blood group,** **N(%)** | A | 189 | 3(24%) | 47(34.8%) | 0.497^‡^ |
|  | AB |  | 16(29.6%) | 30(22.3%) |  |
|  | B |  | 5(9.3%) | 11(8.1%) |  |
|  | O |  | 20(37%) | 47(34.8%) |  |
| **Education, N(%)** | Illiterate | 189 | 3(5.6%) | 18(13.3%) | 0.11^‡^ |
|  | High school |  | 33(61.1%) | 73(54.1%) |  |
|  | Associate |  | 2(3.7%) | 15(11.1%) |  |
|  | Bachelor or above |  | 16(29.6%) | 29(21.5%) |  |
| **Blood Rh, N(%)** | Positive | 189 | 41(75.9%) | 99(73.3%) | 0.71^‡^ |
|  | Negative |  | 13(24.1%) | 36(26.5%) |  |
| **Job, N(%)** | Housewife | 189 | 37(68.5%) | 97(71.9%) | 0.64^‡^ |
|  | Employed |  | 17(31.5%) | 38(28.1%) |  |
| **Pre-existing medical problems, N(%)** | Hypertension | 189 | 5(9.3%) | 19(7.4%) | 0.67^‡^ |
|  | Coronary heart disease | 189 | 0(0%) | 0(0%) | – |
|  | Diabetes | 189 | 2(3.7%) | 7(5.2%) | 0.49^‡^ |
|  | Anemia | 189 | 3(5.6%) | 7(5.2%) | 0.58^§^ |
|  | Hyperthyroidism | 189 | 0 | 0 | – |
|  | Hypothyroidism | 189 | 9(16.7%) | 34(25.5%) | 0.20^‡^ |
|  | pulmonary disease | 189 | 2(3.7%) | 0 | 0.81^§^ |
|  | vitamin D deficiency | 189 | 6(11.1%) | 6(4.4%) | 0.09^‡^ |
|  | Cerebrovascular disease | 189 | 1(1.9%) | 0(0%) | 0.28^§^ |
|  | Liver disease | 189 | 3(5.6%) | 1(7%) | 0.07^§^ |
|  | Immunodeficiency | 189 | 0(0%) | 1(7%) | 0.07^§^ |
|  | Human papilloma virus | 189 | 0(0%) | 1(0.7%) | 0.71^§^ |
|  | Malignancy | 189 | 0(0%) | 2(1.5%) | 0.50^§^ |
|  | Chronic kidney disease | 189 | 0(0%) | 0(0%) | – |
| **Smoking** | Former smoker | 189 | 2(3.7%) | 2(1.5%) | 0.32^§^ |
|  | Current smoker | 189 | 0(0%) | 0(0%) | 0.50^§^ |
| **Drug abuse** | Former drug abuser | 189 | 0(0%) | 3(2.2%) | 0.55^§^ |
|  | Current drug abuser | 189 | 0(0%) | 4(3%) | 0.57^§^ |

^*^The results of Mann Whitney U test, ^†^The result of independent–samples *t* test, ^‡^The result of Chi square test, ^§^The result of Fisher’s exact test
